# Anti-Obesity Effects of Pea Peptides Modified by Steam Explosion on Obese Mice: Regulation of Gut Microbiota and Glucose Metabolism

**DOI:** 10.3390/foods14173008

**Published:** 2025-08-28

**Authors:** Jianqiu Tu, Chenggang Liu, Jingjing Zhang, Tiange Li, Jing Zhu, Qing Wang, Rongrong Wu, Tianlin Wang

**Affiliations:** 1 Research Center for Comprehensive Utilization of Food Resources of Ta-Pieh Mountains, Xinyang Agriculture and Forestry University, Xinyang 464000, China; tujianqiu1022@163.com (J.T.); liuchengang1116@sina.com (C.L.); zhujingcy@163.com (J.Z.); wangqing0118@126.com (Q.W.); 2 College of Food Science and Technology, Henan Agricultural University, Zhengzhou 450002, China; 13126750913@163.com (J.Z.); litiange@henau.edu.cn (T.L.); 3 College of Life Science, Hengshui University, Hengshui 053000, China

**Keywords:** pea peptides, steam explosion, gut microbiota, glucose metabolism, anti-obesity

## Abstract

Pea peptides (PPs), as organic compounds, exhibit a variety of biological functions that make them useful for both the prevention and treatment of metabolic disorders. This study focused on how PPs modified by steam explosion (SE-PP) may help to treat mice with high-fat diet (HFD)-mediated glucose metabolism disorders. The experimental results indicate that both the 100 mg/kg BW SE-PP (SE-PPL group) and 400 mg/kg BW SE-PP (SE-PPH group) experienced substantial decreases in body weight, epididymal and inguinal fat mass, and blood glucose levels of obese mice (notably, the body weight of the SE-PPH group was decreased by 33.13% when compared with that of the HFD group (*p* < 0.05)). By stimulating the IRS-1/PI3K/AKT signaling system, SE-PP controlled glucose metabolism disorder in adipose tissue, while also inhibiting the TLR4/MYD88/NF-κB pathway to reduce inflammation. Furthermore, SE-PP restored the diversity of the gut microbiota destroyed by HFD. SE-PPH increased the *Bacteroidetes*/*Firmicutes* ratio from 0.042 to 0.26 (*p* < 0.05), which is a key indicator of microbiota balance. In addition, SE-PP enhanced the synthesis of short-chain fatty acids (SCFAs) such as isovalerate, propionate, and acetate, which are essential for maintaining intestinal homeostasis and improving metabolic health (supplementation of SE-PPH increased the levels of total SCFAs by 49.87% in obese mice (*p* < 0.05)).

## 1. Introduction

Type 2 diabetes mellitus (T2DM) and cardiovascular illnesses are primarily caused by insulin resistance, which is a major factor in the development of metabolic syndrome [1,2]. Reduced cell sensitivity to insulin is the hallmark of the pathological condition known as insulin resistance, which raises blood glucose levels and causes metabolic problems [3]. Obesity can induce metabolic disorders, damage to signaling pathways, and excessive fat accumulation, all of which are stimulating factors for insulin resistance [4,5]. Research has shown that excessive consumption of high-fat foods, particularly those high in saturated fats, is associated with elevated intracellular blood glucose levels [6,7]. In order to preserve glucose homeostasis, insulin facilitates glucose uptake into the liver, skeletal muscle, and adipose tissue [8]. The primary mechanism for insulin signaling transduction is the IRS/PI3K/AKT signaling pathway [9]. Insulin binding to the insulin receptor (INSR) induces tyrosine residue phosphorylation on insulin receptor substrate (IRS) [10]. IRS further binds to phosphatidylinositol 3-kinase (PI3K) and activates protein kinase B (AKT) to trigger the action of insulin [11]. Insulin resistance may result from impaired PI3K/PIP3/AKT signaling.

Dysbiosis of the gut microbiota induced by high-fat and high-sugar diets can result in insulin resistance. The gut microbiota play an important role in host metabolism and immune regulation [12]. An imbalance in intestinal flora results in changes in the quantity or structure of microbial flora, which undermines the stability of internal and external environments, produces metabolic disorders such as diabetes, and compromises the intestinal barrier’s ability to function [12]. Recent research has revealed that intestinal barrier repair and gut microbiota dysbiosis reversal have emerged as novel targets for insulin signaling activation. An increased *Firmicutes*/*Bacteroides* ratio (F/B) and reduced gut microbiota diversity are closely related to an increased incidence of glucose metabolic disorders, such as type 2 diabetes (T2DM), in hosts [13]. Additionally, a study has demonstrated that the intestinal barrier system is crucial in the intestinal microbiota’s influence on glucose metabolism, and increased intestinal permeability may induce diabetes [14]. Emerging targets for activating insulin signaling pathways have been identified due to the interplay among the gut microbiota, gut barrier integrity, and insulin resistance, alongside efforts to restore microbial balance and repair the gut barrier [15]. Furthermore, intestinal homeostasis and glucose metabolism may be influenced by short-chain fatty acids (SCFAs) [16]. For instance, *Clostridiales* (*Roseburia* and *Faecalibacterium prausnitzii*) produce butyric acid, which can greatly control glucose metabolism [17].

**Figure 1:**
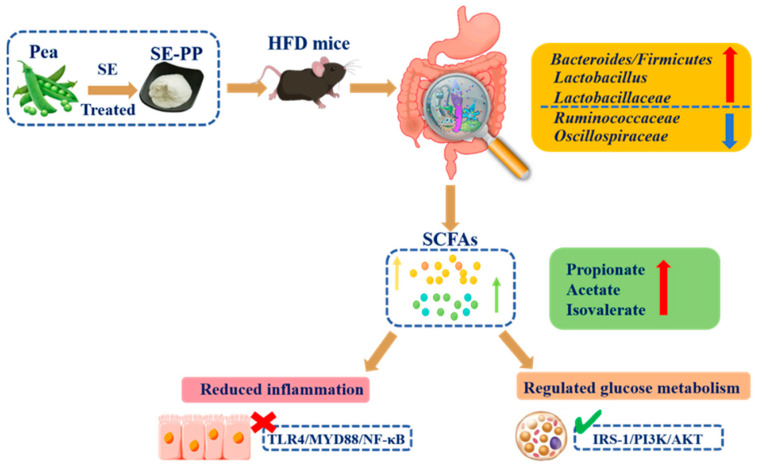
Schematic diagram of SE-PP regulation on gut microbiota and glucose metabolism.

Numerous biological activities are exhibited by the bioactive peptides extracted from pea protein. Pea peptides (PPs), for example, may reduce oxidative stress and inflammation, generate superoxide, and increase the number of vascular smooth muscle cells activated by angiotensin II [18]. PPs have DPP-IV inhibitory activity, thus enhancing glucose tolerance and stimulating glycogen production [19]. In addition, the IRS-1/PI3K/AKT signaling pathway has been shown to be targeted by PPs to reverse IR [20]. Studies have shown that pea protein hydrolysate improves glucose tolerance in T2DM mouse models by inhibiting gluconeogenesis signaling in the liver [21]. As adipose tissue metabolism has become the main determinant of insulin sensitivity throughout the body, various food-derived bioactive peptides have been proven to alleviate metabolic disorders by regulating the insulin signaling pathway in adipose tissue [22,23]. However, there is limited research on the effects of PPs on glucose metabolism in adipose tissue. In addition, steam explosion (SE), an efficient and green physical modification technology, has been widely used to enhance the biological activities of functional factors such as polyphenols, peptides, and dietary fiber [18,24,25]. In our previous study, SE was proven to effectively enhance the lipid-lowering activity of pea peptides in HepG2 cells and in mice fed a high-fat diet (HFD) [26]. Although there are many studies on the hypoglycemic effects of PPs, little information is available on the regulation of glucose metabolism by SE-PP in vivo. Therefore, this study aims to evaluate how PPs modified by steam explosion (SE-PP) influence glucose metabolism in adipose tissue under HFD-induced obesity and assess their influence on modulation of the gut microbiota (Figure 1).

## 2. Materials and Methods

### 2.1. Materials

Pea peptides were purchased form Shanxi Yunhe Biotechnology Co., Ltd. (Xi’an, China). Lysis buffer and BCA protein assay kit were obtained from Beyotime Biotech. Inc. (Shanghai, China). The ELISA kits for INS was obtained from Shanghai Enzyme-linked Biotechnology Co., Ltd. (Shanghai, China). Antibodies against PI3K p110 (T552245), p-PI3K (TA3242), p-IRS-1 (Ser307) (TA3272), phospho-GSK-3 Beta (MA8055) were obtained from Abmart Shanghai Co., Ltd. (Shanghai, China). Antibodies against IRS1 (bs-0172R), phospho-AKT (Ser473) (bs-0876R), TLR4 (bs-20594R), MYD88 (bs-1047R) were obtained from Bioss Antibodies (Beijing, China). Antibodies against GSK3β (22104-1-AP-50), AKT (10176-2-AP), NF-κB p65 (80979-1-RR), ZO-1 (21773-1-AP), Occludin (13409-1-AP) and Tubulin (10094-1-AP) were obtained from Proteintech Group (Wuhan, China).

### 2.2. Preparation of SE-PP

A total of 100 g of pea protein was placed into the reactor chamber of the QBS-80 steam explosion apparatus (Zhengdao Bioenergy Co., Ltd., Hebi, China) with a steam pressure and retention time of 1.2 MPa and 60 s. After this, the steam piston device was activated for explosion decompression, which took place within 0.0875 s. Then, the treated pea protein was hydrolyzed using neutral protease. After inactivation, the SE-PP was obtained by freeze-drying the supernatant.

### 2.3. Animal Experiment Design

The Laboratory Animal Ethics Committee of the Henan Academy of Agricultural Sciences granted approval for all animal protocols (Approval no. LLSC41023028). The mice lived in an environment with a constant temperature of 22 ± 2 °C, 55% humidity, and a 12 h light/dark cycle. Mice were provided with full access to food and water. After adjusting to their new surroundings for a week, male C57BL/6 J mice were categorized into four groups at random: mice fed a control diet (10% kcal from fat, 20% kcal from protein, 70% kcal from carbohydrate, D12450B) (CON group, *n* = 10), mice fed a high-fat diet (60% kcal from fat, 20% kcal from protein, 20 % kcal from carbohydrate, D12492) (HFD group, *n* = 10), mice fed an HFD and 100 mg/kg BW SE-PP (SE-PPL group, *n* = 10), and mice fed an HFD and 400 mg/kg BW SE-PP (SE-PPH group, *n* = 10). The feed formulations are shown in Table S1 in the Supporting Information (SI). The SE-PPL and SE-PPH groups were fed via oral gavage with SE-PP every day for 8 weeks.

### 2.4. Oral Glucose Tolerance Test (OGTT)

Following a 12 h fast, the OGTT was conducted. A blood glucose meter was used to measure the blood glucose levels 0, 30, 60, 90, and 120 min after administering a 20% (*w*/*v*) D-glucose solution (2 g/kg BW, gavage). GraphPad Prism 8.0 was used to determine the area under the curve (AUC) of the OGTT data.

### 2.5. Histological Analysis

For paraffin embedding, the colonic and adipose tissues of mice were preserved in 4% paraformaldehyde. After cutting the samples into 5 μm pieces and staining the wax sections using the hematoxylin and eosin (H&E) method, a light microscope was used to examine the tissue morphology.

### 2.6. Immunohistochemistry

Colon tissues embedded in paraffin were sectioned, and citrate buffer solution was used for antigen repair. After inhibiting endogenous peroxidase activity with a peroxidase-blocking solution, sections were blocked with BSA for an hour at 37 °C. The primary antibody that matched the sections was incubated with them at 4 °C for one night. HRP-conjugated secondary antibodies were specific to the sections. Staining was then examined under a microscope using 100 µL of DAB chromogenic reagent, and hematoxylin was used for nuclear counterstaining. The Image J software (v1.8.0.345) was used for quantitative analysis.

### 2.7. Gut Microbiota Analysis

The total DNA in the cecum was extracted using a DNA kit, and the DNA quantity and quality were verified through agarose gel electrophoresis. Amplification of the V3-V4 regions of the 16S rRNA genes was performed using primers 338F and 806R. For the PCR reactions, initial denaturation was carried out at 95 °C for 5 min; subsequently, 25 cycles were performed (95 °C for 30 s, 50 °C for 30 s, and 72 °C for 40 s). Finally, extension at 72 °C was carried out for 7 min. Purified amplicons were pooled in equimolar amounts and paired-end sequenced on an Illumina MiSeq PE300 platform (reads 300 bp, Illumina, San Diego, CA, USA), according to the standard protocols outlined by Majorbio Bio-Pharm Technology Co., Ltd. (Shanghai, China). The raw sequencing data was processed using the QIIME 1.9.1software for subsequent bioinformatics analysis, including operational taxonomic unit (OTU) clustering, species annotation, microbial community α/β diversity assessment, and correlation analysis.

### 2.8. Untargeted Metabolomics Analysis

The amount of SCFAs in the feces was determined using gas chromatography–mass spectrometry. A 20 mg fecal sample was added to a solution of 0.5% phosphoric acid (containing 10 μg/mL 2-ethylbutyric acid). After freezing and grinding with liquid nitrogen, 10 min of ultrasonic extraction was carried out, followed by 15 min of centrifugation at 13,000× *g*. Then, 200 μL of supernatant and an equal volume of n-butanol were used for liquid–liquid extraction, and sonication was performed for 10 min. After centrifuging again, the supernatant was used for analysis. The program heating conditions were as follows: the initial temperature was 80 °C, which was raised to 120 °C at a rate of 20 °C/min, then raised to 160 °C at a rate of 5 °C/min, and finally maintained at 220 °C for 3 min. The mass spectrometry parameters were as follows: an EI ion source (230 °C), quadrupole (150 °C), transmission line (230 °C), and electron energy (70 eV). The selective ion monitoring mode (SIM) was used. The standard curve prepared for each standard reagent was used to calculate the concentrations of each acid. The calibration curves (R2 values) and limits of detection are shown in Table S2.

### 2.9. Western Blot Analysis

Total protein was extracted from adipose and colon tissue by mixing 300 mg of adipose tissue and 100 mg of colon tissue with 1000 μL of RIPA buffer. After completely grinding the mixture on ice, it was centrifuged for 15 min (4 °C, 12,000 r/min). To measure protein levels, the BCA assay kit was employed (Beyotime, China). After separating proteins using a 10% SDS-PAGE, they were transferred to polyvinylidene difluoride membranes (Millipore, Boston, MA, USA). After blocking the membranes at room temperature for one hour with 5% skim milk to allow binding, the samples were incubated overnight at 4 °C with primary antibodies. Fluorescent secondary antibody incubation was carried out in the dark at room temperature for one additional hour, after which the samples were washed five times with TBST. An image was captured using a gel imager, and the Image J software was used to perform grayscale analysis.

### 2.10. Statistical Analysis

The statistical analysis was conducted using GraphPad Prism 8.0 and SPSS 20.0. The sample size was determined through power calculation. Group variance was analyzed with an F test. Data are reported as the mean ± standard error, and group comparisons were made using a one-way ANOVA. In the case of homogeneity of variances, Duncan’s multiple range test (multiple F test) was used to identify any difference in mean values. A significant difference between groups (*p* < 0.05) is shown by different letters; the same letters indicate no statistical difference.

## 3. Results

### 3.1. The Effect of SE-PP on Body Weight, Fat Mass Index and Blood Glucose of Mice

**Figure 2:**
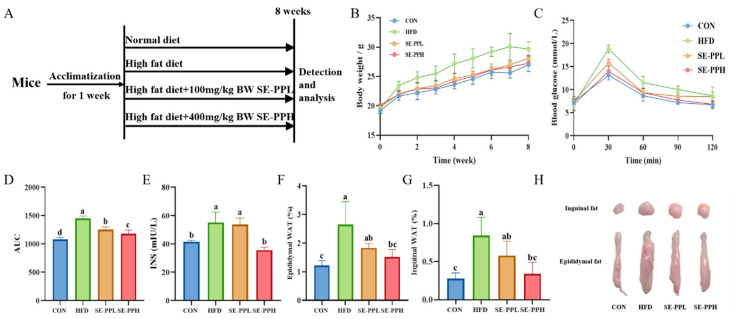
SE-PP attenuates weight gain and enhances glucose homeostasis in HFD-induced obese mice. (**A**) Experimental protocol and design. (**B**) Body weight. (**C**) OGTT. (**D**) Area under the curve (AUC) analysis of OGTT, (**E**) insulin (INS) levels, (**F**) epididymal WAT, (**G**) inguinal WAT; (**H**) representative photographs of epididymal and inguinal fat. Data are means ± SEM (n = 7–8 per group). Different letters reflect significant differences between groups (*p* < 0.05), while the same letters denote no significant differences. Data are means ± SEM (n = 7–8 per group).

In Figure S1, after SE treatment, the relative content of pea peptides < 3 kDa were increased, while the relative content of pea peptides > 10 kDa were declined, indicating SE leads to the formation of more small-molecule peptides. However, the contents of main amino acids were similar between PP and SE-PP (Figure S2). The major amino acids were Phe and Tyr, followed by Cys, Leu, and Glu. The contents of Tyr, Leu, and Phe were decreased after SE treatment. Figure 2A shows a simplified flowchart of animal experiments, and Figure 2B shows the body weight changes in all groups of mice throughout the entire experimental period. The body weight of the HFD group was significantly greater than that of the CON group. Nonetheless, body weight was considerably decreased by SE-PP intervention. As shown in Figure S3, the energy intake did not significantly differ among the four groups during the intervention period, indicating the effect of SE-PP on reducing body weight gain was not due to changes in energy intake. Following glucose gavage, the blood glucose levels in each group peaked at 30 min, as shown in Figure 2C. Furthermore, the HFD group exhibited persistently higher blood glucose levels than all other groups. In contrast to the HFD group, the OGTT and AUC values in the SE-PPL and SE-PPH groups (Figure 2D) were noticeably lower. Compared to the CON group, the INS levels (Figure 2E) in the HFD group increased. After the SE-PP intervention, the mice showed a decrease in INS levels based on different sample concentrations. Furthermore, as shown in Figure 2F–H, the SE-PP intervention considerably decreased the fat weight of the mice, while the weights of inguinal and epididymal fat in the HFD group increased significantly compared to the CON group. Furthermore, the high-dose intervention yielded similar results to the CON group. These results indicate that the SE-PP treatment significantly reduced blood glucose levels in mice.

### 3.2. The Effect of SE-PP on Insulin Resistance and Inflammatory Response in Adipose Tissue Induced by HFD

**Figure 3:**
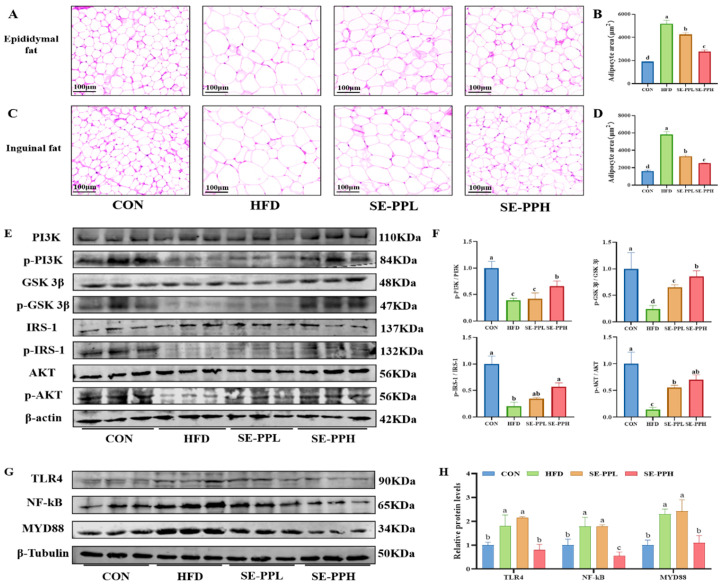
SE-PP attenuates insulin resistance in HFD-induced obese mice. (**A**) Representative H&E staining of epididymal fat. (**B**) Adipocyte area of epididymal fat tissue (μm^2^). (**C**) Representative H&E staining of inguinal fat. (**D**) Adipocyte area of inguinal fat tissue (μm^2^). (**E**) Western blot images of PI3K, p-PI3K, GSK3β, p-GSK3β, IRS-1, p-IRS-1, AKT, p-AKT. (**F**) Protein expressions of p-PI3K/PI3K, p-GSK3β/GSK3β, p-IRS-1/IRS-1, p-AKT/AKT. (**G**) Western blot images of TLR4, MYD88, and NF-κB. (**H**) Protein expressions of TLR4, MYD88, and NF-κB. Data are means ± SEM (n = 6 per group). Different letters reflect significant differences between groups (*p* < 0.05), while the same letters denote no significant differences.

As shown in Figure 3A–D, H&E staining indicated that the size of adipose tissue cells in the HFD group was significantly larger than in the CON group, while SE-PP administration reduced the size of cells in both epididymal (Figure 3A,B) and inguinal (Figure 3C,D) adipose tissue. In addition, Figure 3E,F show that the HFD group experienced significantly downregulated phosphorylation levels of the key sugar utilization and regulation-related proteins PI3K, GSK 3β, IRS-1, and AKT in adipose tissue. However, the expression of these proteins significantly increased after the SE-PP intervention, and the results for the SE-PPH group were closer to those of the CON group. Therefore, SE-PP can improve HFD-induced insulin resistance in adipose tissue. In addition, the effect of SE-PP on inflammation in adipose tissue was measured through the NF-kB pathway. As shown in Figure 3G,H, compared with the CON group, an HFD induced significant activation of TLR4/MyD88/NF-κB protein expression. However, following the SE-PP intervention, a substantial reduction in TLR4, MyD88, and NF-κB expression was detected when compared to the HFD group. Similar protein expression patterns were found in both the SE-PPH and CON groups.

### 3.3. The Effect of SE-PP on Gut Dysbiosis Induced by an HFD

**Figure 4:**
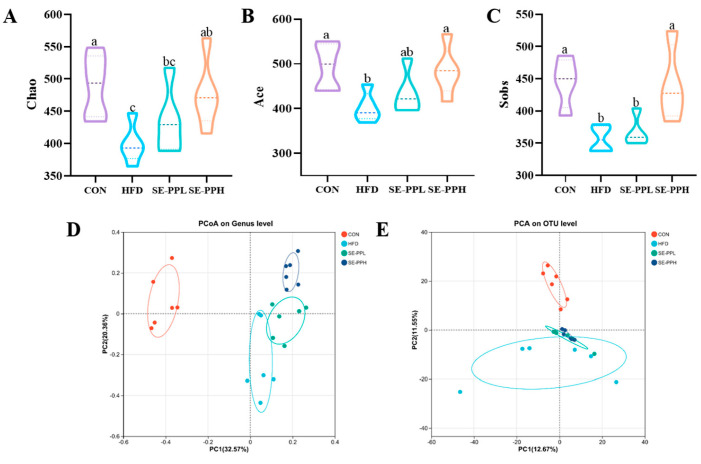
The gut microbiota structure in HFD-induced obese mice is regulated by SE-PP. α-Diversity analysis including (**A**) Chao index, (**B**) Ace index, (**C**) Sobs index. (**D**) PCoA analysis on Genus level, and (**E**) PCA analysis on OTU level. Data are means ± SEM (n = 6–7 per group). Different letters reflect significant differences between groups (*p* < 0.05), while the same letters denote no significant differences.

The gut microbiota response to SE-PP was investigated through 16S rRNA high-throughput sequencing of cecal samples from various mouse groups. The microbial species diversity in the sample was assessed using the alpha diversity, which includes the Chao, Ace, and Sobs indices. These indices were significantly lower in the HFD group, as seen in Figure 4A–C. This suggests that the HFD drastically decreased microbial diversity and fecal microbiota richness. However, in mice with HFD-induced obesity, recovery in gut microbial richness and diversity was observed after SE-PP administration. The results of the principal coordinate analysis (PCoA) (Figure 4D) and principal component analysis (PCA) (Figure 4E) demonstrated a substantial difference between the HFD and CON groups, suggesting that the gut microbiota structure was significantly altered by the HFD. However, the groups that were administered different doses of the SE-PP intervention were separated from the HFD group and clustered more with the CON group in the PC2 direction, indicating that the gut microbiota structure altered by the HFD was effectively shifted by SE-PP in the direction of the CON group’s profile.

**Figure 5:**
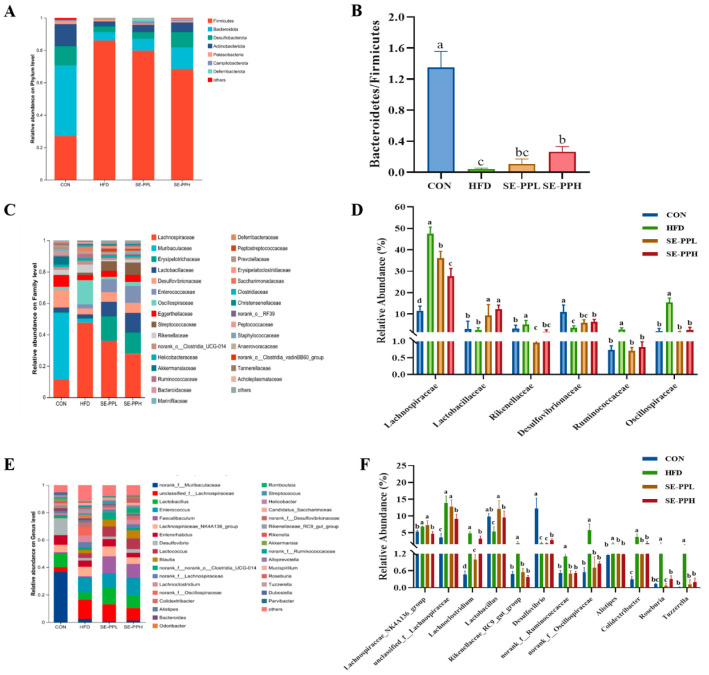
The composition of the gut microbiota in obese mice induced by HFD is modified by SE-PP. (**A**) A bar chart showing the composition at the phylum level for gut microbiota. (**B**) Relative proportion of sequence at the phylum level. (**C**) A family-level bar plot demonstrating the makeup of the gut microbiota. (**D**) Relative proportion of sequence in family level. (**E**) A bar graph showing the composition of gut microbiota at the genus level. (**F**) Relative proportion of sequence in genus level. Data are means ± SEM (n = 6–7 per group). Different letters reflect significant differences between groups (*p* < 0.05), while the same letters denote no significant differences.

Furthermore, the composition of intestinal flora was compared by looking at the amount of bacteria at the phylum, family, and genus levels. As shown in Figure 5A,B and Table S3, the HFD group had a considerably decreased *Bacteroidetes*/*Firmicutes* (B/F) ratio at the phylum level. Following the SE-PP intervention, there was a notable rise in the B/F ratio. The HFD group had a significantly higher relative abundance of *Lachnospiraceae*, *Rikenellaceae*, *Ruminococcaceae*, and *Oscillospiraceae* than the CON group, whereas *Lactobacillaceae* and *Desulfovibrionaceae* had significantly lower relative abundances, as seen in Figure 5C,D and Table S4. However, the SE-PP intervention increased the abundances of *Lactobacillaceae* and *Desulfovibrionaceae* while decreasing the relative abundances of *Lachnospiraceae*, *Rikenellaceae*, *Ruminococcaceae*, and *Oscillospiraceae*. At the genus level, the relative abundances of *Lachnoclostridium*, *Rikenellaceae*_RC9_gut_group, norank_f_*Ruminococcaceae*, norank_f_*Oscillospiraceae*, *Alistipes*, *Colidextribacter*, *Roseburia*, and *Tuzzerella* were significantly increased, while those of *Lactobacillus* and *Desulfovibrio* were decreased, as shown in Figure 5E,F and Table S5. The gut microbiota’s response to the HFD was neutralized through the SE-PP intervention. Increases in *Lachnoclostridium*, *Rikenellaceae*_RC9_gut_group, norank_f_*Ruminococcaceae*, norank_f_*Oscillospiraceae*, *Alistipes*, *Colidextribacter*, *Roseburia*, and *Tuzzerella* and clear avoidance occurred with the decline in the relative abundances of *Lactobacillus* and *Desulfovibrio*.

To explore the correlation between serum biochemical indices (including inflammatory factors and metabolic parameters) and specific bacterial taxa at the genus level, Spearman’s rho nonparametric correlation heatmap was tabulated. These indices include TG, TC, HDL-C, LDL-C, INS, LPS, IL-6, and TNF-α. Figure S4 shows that *Lachnoclostridium* is positively correlated with TC and INS. norank_f_*Ruminococcaceae* had a positive relationship with IL-6, INS, LPS, and TNF-α. Tuzzerella is positively correlated with TC, LCL-C, IL-6, INS, LPS, and TNF-α. In addition, *Lachnoclostridium*, *Rikenellacea*_RC9_gut_group, norank_f_*Ruminococcaceae*, norank_f_*Oscillospiraceae*, *Colidextribacter*, *Roseburia*, and *Tuzzerella* are all negatively correlated with HDL-C.

### 3.4. The Effect of SE-PP on Gut Microbial Metabolic Functions

**Figure 6:**
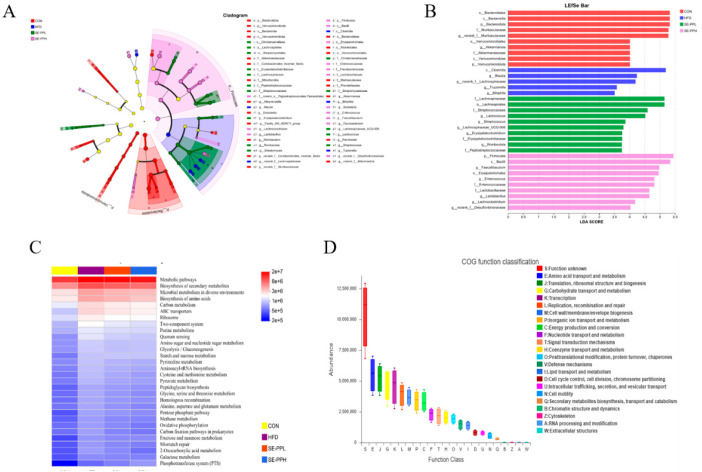
Predicted metabolic functions of gut microbiota. (**A**) LEfSe analyses of gut microbiota. (**B**) Features at the OTU level are represented by LDA scores in the taxonomic histograms. The taxa that are differentially expressed have LDA scores greater than 2.0 and p values less than 0.05. (**C**) The role of KEGG pathways in microbial metabolism at level 3. (**D**) COG function classification analyses.

The microbiota and their taxonomic hierarchies were illustrated using a histogram of LDA value distribution and an associated cladogram (LDA score > 2.0). As shown in Figure 6A,B, significant separation of gut microbiota was observed among the four groups. In addition, to determine whether SE-PP affects the function of microbial communities, predictions of gut microbiota metabolic characteristics were made from the relative abundance of KEGG pathways using PICRUSt2 analysis. Figure 6C,D show that at the pathway 3 level, the amino acid, carbohydrate, and lipid metabolism pathways were the main factors associated with the intestinal flora changes in each group.

### 3.5. The Effect of SE-PP on SCFA Profiles in Mice Induced with the HFD

**Figure 7:**
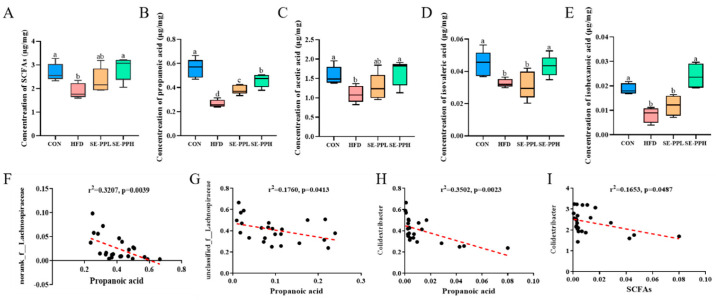
SE-PP improved SCFAs in HFD-induced obese mice. Concentrations of (**A**) SCFAs, (**B**) propanoic acid, (**C**) acetic acid, (**D**) isovaleric acid, and (**E**) isohexanoic acid. The correlation between propanoic acid and (**F**) norank_f_Lachnospiraceae, (**G**) unclassified_f_Lachnospiraceae, (**H**) Colidextribacter, and (**I**) SCFAs. Data are means ± SEM (n = 6 per group). Different letters reflect significant differences between groups (*p* < 0.05), while the same letters denote no significant differences.

To evaluate the effect of SE-PP on the spectrum of SCFAs, we detected the contents of SCFAs in the ceca of mice. A significant reduction in SCFA production occurred in the HFD group relative to the CON group, as illustrated in Figure 7A–E. However, high and medium doses of SE-PP supplementation led to remarkably higher levels of propanoic acid, acetic acid, isovaleric acid, and isohexanoic acid than in the HFD group. According to these findings, SE-PP may help HFD mice’s SCFA profiles to better resemble those of healthy mice. As shown in Figure 7F–I, at the genus level, propanoic acid was negatively correlated with norank_f_*Lachnospiraceae*, unclassified_f_*Lachnospiraceae*, and *Colidextribacter*. These findings indicate that SE-PP potentially modulates gut microbiota and their metabolites, including SCFAs, to improve glycometabolic function and related disorders.

### 3.6. The Effect of SE-PP on Gut Inflammation in Mice Induced by an HFD

**Figure 8:**
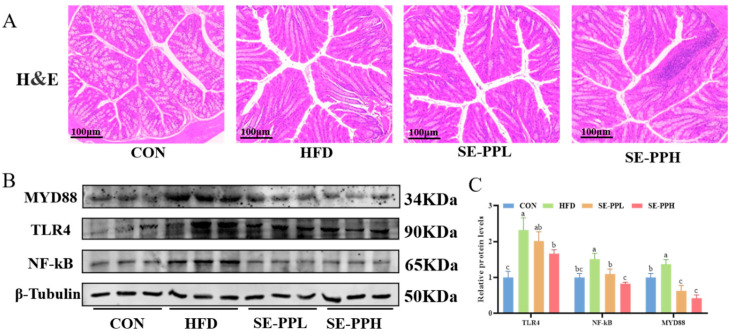
SE-PP regulates intestinal inflammation in HFD-induced obese mice. (**A**) H&E-stained sections of colonic tissue. (**B**) Western blot images of MYD88, TLR4, and NF-κB. (**C**) Protein expressions of MYD88, TLR4, and NF-κB. Data are means ± SEM (n = 6 per group). Different letters reflect significant differences between groups (*p* < 0.05), while the same letters denote no significant differences.

As shown in Figure 8A, normal tissue structure was observed in the CON group through H&E staining. In contrast, the HFD group displayed histological damage with damaged gut mucosa and inflammatory infiltration. However, pathological damage was significantly reduced after the SE-PPL and SE-PPH treatments. To determine the extent to which SE-PP reduces intestinal tissue damage, the expression of inflammatory mediators at the molecular level in the gut tissue was quantified. Figure 8B,C illustrate how the HFD significantly increased the colon’s MYD88, TLR4, and NF-κB protein expression compared with the CON group. However, the expression of MYD88, TLR4, and NF-κB was decreased by SE-PP in a dose-dependent manner, suggesting its role in regulating colonic inflammation through the TLR4/MYD88/NF-κB pathway.

**Figure 9:**
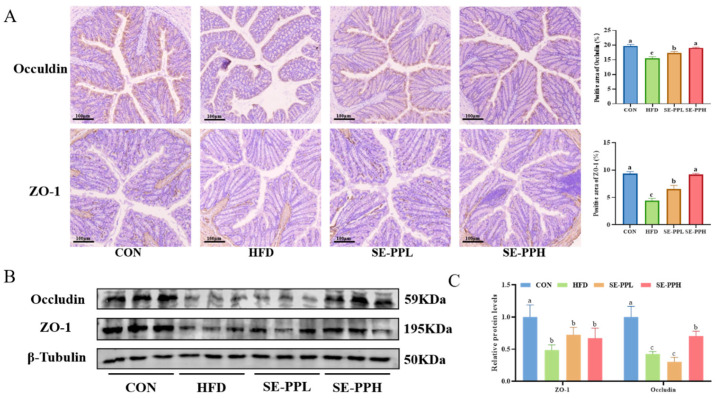
SE-PP regulates the intestinal barrier in HFD-induced obese mice. (**A**) Immunohistochemical staining of Occludin and ZO-1. (**B**) Western blot images of Occludin and ZO-1. (**C**) Occludin and ZO-1 protein expressions are presented. Different letters reflect significant differences between groups (*p* < 0.05), while the same letters denote no significant differences.

Furthermore, the effects of SE-PP on intestinal barrier function were investigated. Using immunohistochemistry, the expression and localization of tight-binding proteins (Occludin and ZO-1) were assessed. Figure 9A shows that the expression of Occludin and ZO-1 in the HFD group was significantly decreased compared to the CON group. However, SE-PPL and SE-PPH treatments increased the expression of ZO-1 and Occludin proteins. Furthermore, the expression levels of ZO-1 and Occludin were measured via Western blot, as shown in Figure 9B,C. The immunohistochemistry findings of tight-binding proteins are in accordance with the restoration of ZO-1 and Occludin expression by SE-PPL and SE-PPH, suggesting that SE-PP improved the colon’s intestinal barrier.

## 4. Discussion

The associations between insulin resistance, a high-fat diet, and obesity have become a growing focus of research [3]. A diet heavy in fat may make insulin less effective through dysfunctional insulin secretion, reduced glucose tolerance, increased content of cellular fat, and reduced insulin sensitivity in peripheral tissue [27]. In this study, pea peptides were prepared by steam explosion-assisted enzymatic hydrolysis. The administration of pea peptides reduced weight gain and insulin resistance in the adipose tissue of mice with high-fat-diet-induced obesity; additionally, improvements in gut microbiota composition and function were observed, along with a rise in short-chain fatty acid levels in microbial metabolites.

The levels of FBG, INS, and OGTT are key evaluation indicators for the diagnosis and treatment of diabetes patients [28], reflecting the control of the body’s basic blood sugar levels [29]. Our results indicate that the levels of FBG, INS, and AUC were significantly reduced after the pea peptide treatment, which is consistent with a previous study [30]. Beyond its role in energy storage, systemic metabolism is regulated in part through signals sent by adipose tissue to other organs such as the liver, brain, pancreas, and muscles [31]. Numerous adipokines, including adiponectin, leptin, and resistin, are secreted by adipose cells and can result in metabolic disorders and systemic inflammation [32]. In adipose tissue, insulin-stimulated glucose absorption mostly relies on IRS-1-PI3K-AKT pathway activation [33]. An essential function of this signaling pathway is controlling glucose metabolism, including glycogen synthesis, promoting glucose uptake, and inhibiting gluconeogenesis [33]. The results indicate that the expression of glucose metabolism related to the proteins PI3K, AKT, IRS-1, and GSK 3β was increased after the pea peptide intervention in adipose tissue. Walnut peptides reduced gluconeogenesis by activating the IRS-1/PI3K/Akt/GSK 3β signaling pathway [34]. In summary, treatment with pea peptides can effectively reduce blood glucose levels and glucose tolerance in mice with high-fat-diet-induced obesity, regulating glucose metabolism through the IRS-1/PI3K/Akt signaling pathway.

Studies have demonstrated that the development of type 2 diabetes mellitus (T2DM) is largely influenced by an imbalance in the gut microbiota [35]. Changes in gut microbiota composition and diversity may be responsible for this outcome. In obese mice, α-diversity and β-diversity are tools to measure microbial richness and community structure [36]. In our study, the HFD significantly reduced the α-diversity of the gut microbiota in obese mice, including the Chao, Ace, and Sobs indices. These findings are comparable to previous studies [37,38], indicating that an HFD may disrupt the balance of gut microbial communities, ultimately affecting metabolic health. However, intervention with SE-PP significantly restored the α-diversity of gut microbiota in obese mice. In particular, the SE-PPH group showed diversity levels similar to those of the control group. This suggests that SE-PP can effectively improve the reduction in gut microbiota diversity caused by an HFD, possibly by promoting the growth of beneficial bacteria or suppressing harmful ones. Additionally, β-diversity analysis revealed that the SE-PP intervention substantially modified the gut microbiota’s composition, making it more similar to the control group. This indicates that SE-PP can regulate the structure of the gut microbiota.

Disturbances in glucose metabolism can lead to disruptions in the composition of the gut microbiota, demonstrating their correlation [39]. A low *Bacteroidetes* to *Firmicutes* (B/F) ratio is widely recognized as a sign of gut microbiota imbalance [40,41]. A decreased B/F ratio is regarded as dysbiosis, which is usually observed with obesity [42]. Similarly to previous research results, the HFD decreased the B/F ratio in this study, while treatment with SE-PP significantly increased the B/F ratio to restore the gut microbiota’s balance. We found that the B/F ratio was also increased by α-lactalbumin peptides, thus improving insulin resistance and alleviating gut microbiota dysbiosis in HFD mice [43]. *Ruminococcaceae* is one of the most abundant microbial families in the intestine, with extensive carbohydrate-degrading activity [40]. *Ruminococcaceae* promotes fat synthesis [44], and the relative abundance of *Ruminococcaceae* is closely related to an HFD [45]. In our study, treatment with SE-PP reduced the relative abundance of *Ruminococcaceae*. Consistent with this, Rosa *roxburghii* Tratt fruit vinegar significantly reduced the abundance of *Ruminococcaceae* in mice fed a high-fat diet, thereby improving dyslipidemia [45]. The abundance of *Oscillospiraceae* was increased with the rise in BMI, and it was also associated with an increased risk of T2DM [46]. One study showed that Macauba (*Acrocomia aculeata*) pulp oil has the potential to enhance the gut microbiota composition in mice fed an HFD, with a decrease in *Oscillospiraceae* abundance [47]. Consistent with the above results, the HFD group showed a higher relative abundance of *Oscillospiraceae*, while SE-PP treatment decreased the abundance of *Oscillospiraceae* in HFD mice in the present study. *Lactobacillaceae* are beneficial bacteria that produce lactic acid and SCFAs, lower the pH value of the intestine, and maintain intestinal homeostasis [48]. The relative abundances of *Lactobacillus* and *Lactobacillaceae* were increased by SE-PP. Similarly to our findings, other reports have also shown that active peptides from *Eupolyphaga sinensis* changed the gut microbiota composition and increased the relative abundance of beneficial bacteria, including *Lactobacillus* [49]. Adzuki bean protein hydrolysates enriched *Lactobacillus* and SCFA-producing bacteria and regulated the levels of *Lactobacillus*-derived metabolites, which help to alleviate obesity and its complications [44]. In summary, these results suggest that SE-PP can maintain intestinal balance by regulating the gut microbiota.

Short chain fatty acids (SCFAs) are important products of gut microbiota metabolism, which can promote the growth of beneficial bacteria and maintain intestinal health [50]. SCFAs can reduce obesity and improve insulin sensitivity. In this study, intervention with SE-PP significantly increased the total SCFA content in obese mice, particularly propionate, acetate, and isovalerate levels. Cheng’s group found that the structure of the intestinal microbiota in mice was modulated by black bean peptides, which enhanced beneficial bacterial populations, reduced harmful bacteria, and increased SCFA levels, thereby maintaining gut microbiota homeostasis [51]. The outcomes of this investigation are in line with these previous findings, indicating that SE-PP has the potential to modulate the metabolic functions of intestinal microbiota and stimulate the production of SCFAs.

In addition, the structure of peptides influences their biological activities. Studies have shown that certain hydrophilic amino acids (such as cysteine and arginine), hydrophobic amino acids (such as leucine, phenylalanine, proline, and glycine), and aromatic amino acids (such as tryptophan and tyrosine) play significant roles in the alleviation of obesity and glucose metabolism disorders [52]. For instance, they can inhibit the activity of α-amylase through hydrophobic interactions and hydrogen bonds, thereby reducing the hydrolysis of polysaccharides and lowering blood glucose levels [53]. However, the structural characteristics of highly active anti-obesity or anti-diabetic bioactive peptides are still limited. As the pea peptides used in this study were mixtures, it is not clear which peptides play a decisive role. Therefore, future research can further study the separation and purification of pea peptides treated by SE in order to explore and verify the associated mechanisms.

## 5. Conclusions

This study showed that SE-PP effectively alleviated glucose metabolism disorder in mice with HFD-induced obesity by regulating adipose tissue IR and intestinal microbiota. By activating the IRS-1/PI3K/AKT signaling pathway in adipose tissue, SE-PP reduced IR. Likewise, it inhibited inflammation by modulating TLR4/MYD88/NF-κB signaling. In addition, the composition and diversity of the gut microbiota improved, and SCFA levels were elevated following SE-PP treatment. This study presents a new idea and theoretical basis for the prevention, improvement, and dietary intervention of T2DM. However, this study has certain limitations, such as the lack of a long-term safety assessment. Additionally, issues such as the absorption of peptides in the gastrointestinal tract and individual variability in the response to bioactive peptides need further investigation. Therefore, more research—especially clinical trials—is needed to assess the efficacy of SE-PP in the prevention and treatment of metabolic disorders.

## Data Availability

The original contributions presented in the study are included in the article/Supplementary Materials, further inquiries can be directed to the corresponding authors.

## Supplementary Materials

The following supporting information can be downloaded at: https://www.mdpi.com/article/10.3390/foods14173008/s1, Figure S1: Molecular mass distribution of PP and SE-PP; Figure S2: Amino acids contents of PP and SE-PP; Figure S3: Energy intake of mice; Figure S4: Correlation between the obesity-related index and gut bacteria (the relative abundances of 50 key phylotypes) at the genus level. The color of cells represents correlation R values from (−1)–0–1 (blue–white–red); Table S1: The feed formulation; Table S2: Calibration curves for untargeted metabolomics analysis; Table S3: The relative percentages of the gut microbiota at the phylum level; Table S4: The relative percentages of the gut microbiota at the family level; Table S5: The relative percentages of the gut microbiota at the genus level.
