# Supplementary material for: Anti-Obesity Effects of Pea Peptides Modified by Steam Explosion on Obese Mice: Regulation of Gut Microbiota and Glucose Metabolism
Source: Foods. 2025 Aug 28;14(17):3008. doi: 10.3390/foods14173008 (PMC12428707; doi:10.3390/foods14173008)
Supplement: Supplementary file 1 [file foods-14-03008-s001.zip › foods-3827666-supplementary.pdf]

# Supporting information

# Anti-obesity effects of pea peptides modified by steam explosion on obese mice: Regulation of gut microbiota and glucose metabolism

Jianqiu Tu [1], Chenggang Liu [1], Jingjing Zhang [2], Tiange Li [2], Jing Zhu [1], Qing Wang [1], Rongrong Wu [3*], Tianlin Wang [2*]

[1]Research Center for Comprehensive Utilization of Food Resources of Ta-pieh Mountains, Xinyang Ag-riculture and Forestry University, Xinyang 464000, China. tujianqiu1022@163.com (J. Tu); liuchengang1116@sina.com (C. Liu); zhujingcy@163.com (J. Zhu); wangqing0118@126.com (Q. Wang)

[2]College of Food Science and Technology, Henan Agricultural University, Zhengzhou 450002, China. 304234443@qq.com (J. Zhang); litiange@henau.edu.cn (T. Li); wangtianlin@henau.edu.cn (T. Wang)

[3] College of Life Science, Hengshui University, Hengshui 053000, China. rpkxw@126.com (R. Wu)

*Correspondence: rpkxw@126.com (R. Wu); wangtianlin@henau.edu.cn (T. Wang)

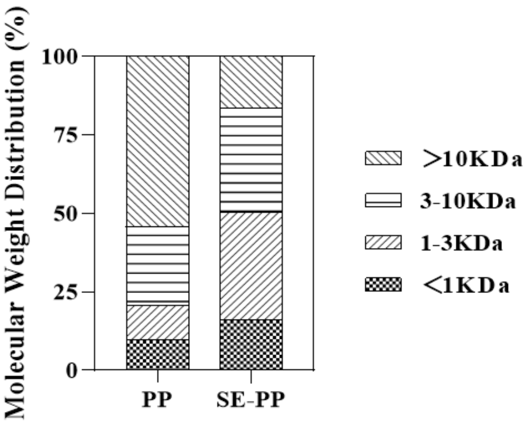


**Figure S1** Molecular mass distribution of PP and SE-PP

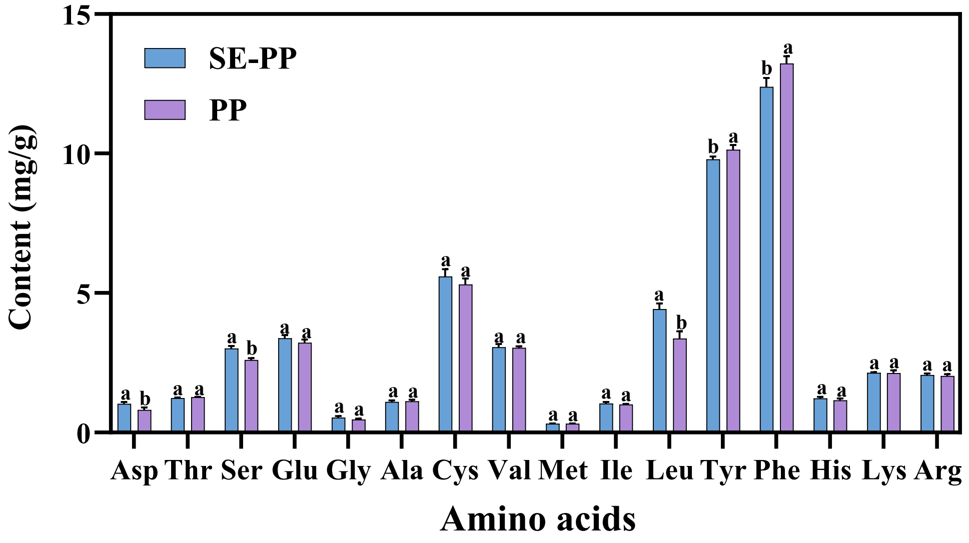


**Figure S2** Amino acids contents of PP and SE-PP

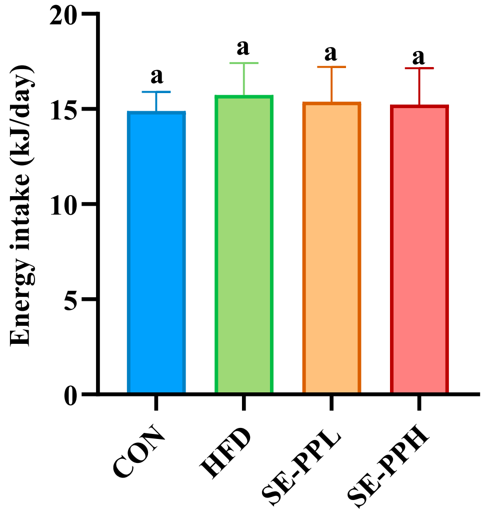


**Figure S3** Energy intake of mice

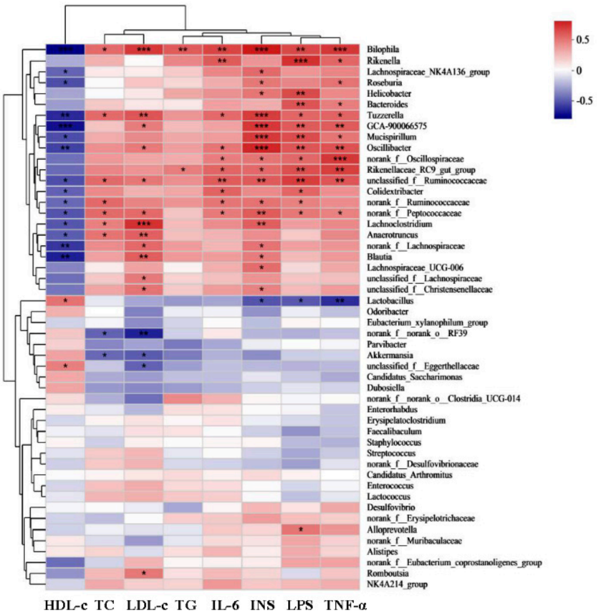


**Figure S4** Correlation between the obesity-related index and gut bacteria (the relative abundances of 50 key phylotypes) at the genus level. The color of cells represents correlation R values from (−1)–0–1 (blue–white–red).

**Table S1 The feed formulation**

| | D12450B | | D12492 | |
|---|---|---|---|---|
| Ingredient | gm | Kcal | gm | Kcal |
| Casein, 80 Mesh | 200 | 800 | 200 | 800 |
| L-Cystine | 3 | 12 | 3 | 12 |
| Corn Starch | 315 | 1260 | 125 | 500 |
| Maltodextrin 10 | 35 | 140 | 68.8 | 275.2 |
| Sucrose | 350 | 1400 | 50 | 225 |
| Cellulose, BW200 | 50 | 225 | 25 | 2205 |
| Soybean Oil | 25 | 180 | 245 | 40 |
| Lard* | 20 | 40 | 10 | 0 |
| Mineral Mix S10026 | 10 | 0 | 13 | |
| DiCalcium Phosphate | 13 | | 5.5 | |
| Calcium Carbonate | 5.5 | | 16.5 | |

| | | | | |
|---|---|---|---|---|
| Potassium Citrate, 1 $H_2O$ | 16.5 | | 10 | |
| Vitamin Mix V1001 | 10 | | 2 | |
| Choline Bitartrate | 2 | | 0.05 | |
| FD&C Yellow Dye #5 | 0.05 | | | |
| FD&C Red Dye #40 | | | | |
| FD&C Blue Dye #1 | | | | |
| Total | 1055.05 | 4057 | 773.85 | 4057 |

**Table S2** Calibration curves for untargeted metabolomics analysis

| name | range (μg/mL) | calibration curves | $R^2$ | limits of detection |
|---|---|---|---|---|
| Acetic acid | 0.48-600 | y = 0.490132 * x+0.042367 | 0.999 | 2.4 |
| Propanoic acid | 0.24-300 | y = 0.490948 * x+0.004762 | 0.999 | 1.2 |
| Butanoic acid | 0.04-50 | y = 1.319413 * x+0.006782 | 0.998 | 0.52 |
| Isobutyric acid | 0.04-50 | y = 0.984648 * x+0.006580 | 0.998 | 0.60 |
| Valeric acid | 0.04-50 | y = 1.444959 * x- 9.704039E-005 | 0.996 | 0.40 |
| Isovaleric acid | 0.08-100 | y = 1.602470 * x- 0.001425 | 0.998 | 0.52 |
| Hexanoic acid | 0.04--50 | y = 1.221868 * x- 0.001199 | 0.999 | 0.40 |
| Isohexanoic acid | 0.04-50 | y = 0.610351 * x+ 7.376862E-004 | 0.997 | 0.40 |

**Table S3** The relative percentages of the gut microbiota at the phylum level

| name | CON | HFD | SE-PPL | SE-PPH |
|---|---|---|---|---|
| *Firmicutes* | 0.270369070875 | 0.860555313217 | 0.7969825706 | 0.68413715933 |
| *Bacteroidota* | 0.436133290345 | 0.0510127638591 | 0.0748288353216 | 0.134295522218 |
| *Desulfobacterota* | 0.11827431866 | 0.0353139757619 | 0.0412359891517 | 0.094425057371 |
| *Actinobacteriota* | 0.136566654655 | 0.0311605060026 | 0.0445454890284 | 0.0596847914731 |
| *Patescibacteria* | 0.0180694900146 | 0.0111754888387 | 0.00477459366169 | 0.00795133423104 |
| *Campilobacterota* | 0.00826426688414 | 0.00322889601153 | 0.0175052629583 | 0.00759572894343 |
| *Deferribacterota* | 0.000113793692036 | 0.00459442031597 | 0.0186242342633 | 0.00721641663664 |
| others | 0.0122091148747 | 0.00295863599294 | 0.00150302501565 | 0.0046939897965 |

**Table S4** The relative percentages of the gut microbiota at the family level

| name | CON | HFD | SE-PPL | SE-PPH |
|---|---|---|---|---|
| *Lachnospiraceae* | 0.115902668462 | 0.476454188556 | 0.361420145277 | 0.276241773664 |
| *Muribaculaceae* | 0.421082178011 | 0.0242285735961 | 0.00175621598043 | 0.00371346748345 |
| *Erysipelotrichaceae* | 0.00438485026647 | 0.00357027708764 | 0.152840100897 | 0.134090693573 |
| *Lactobacillaceae* | 0.0311111954027 | 0.0250251294403 | 0.0929011701785 | 0.124114779904 |
| *Desulfovibrionaceae* | 0.111130919643 | 0.0369971741233 | 0.0602803117947 | 0.0637927437556 |
| *Enterococcaceae* | 0.000891383920951 | 0.0262816014566 | 0.0828114628179 | 0.107273313483 |
| *Oscillospiraceae* | 0.0199594135832 | 0.153924934094 | 0.0154493902555 | 0.0254290970471 |
| *Eggerthellaceae* | 0.0740721072695 | 0.0321609422118 | 0.0380298518785 | 0.0452216132152 |
| *Streptococcaceae* | 0.0018662165494 | 0.0154806835208 | 0.0608947977317 | 0.0790676503499 |
| *Rikenellaceae* | 0.0320632692928 | 0.0523830295674 | 0.00986211997648 | 0.0203918296129 |

| | | | | |
|---|---|---|---|---|
| norank_o__*Clostridia*_UCG-014 | 0.0182600944488 | 0.0239867620005 | 0.014080072828 | 0.012517306124 |
| *Helicobacteraceae* | 0.0155214595938 | 0.0151061126178 | 0.0146528344112 | 0.0109962637738 |
| *Akkermansiaceae* | 0.0510592296167 | —— | —— | 0.00311036091566 |
| *Ruminococcaceae* | 0.00748383181292 | 0.028680751797 | 0.00730176190567 | 0.00828038765718 |
| *Bacteroidaceae* | 0.0251939234168 | 0.0143000739659 | 0.00498037058812 | 0.0039562273598 |
| *Marinifilaceae* | 0.01195592391 | 0.00293967037761 | 0.00632313615415 | 0.0153355965634 |
| *Deferribacteraceae* | 0.00192311339541 | 0.024489350807 | 0.00475657632712 | 0.00168793976521 |
| *Peptostreptococcaceae* | 0.0000948280766969 | 0.000256035807082 | 0.0194738938305 | 0.0114286798035 |
| *Prevotellaceae* | 0.0198873442449 | 0.00308665389649 | 0.00183207844179 | 0.00201414834904 |
| *Erysipelatoclostridiaceae* | 0.00274622110114 | 0.000777590228915 | 0.0123352362167 | 0.00726003755192 |
| *Saccharimonadaceae* | 0.00644830921539 | 0.00139397272745 | 0.00385381303696 | 0.00815521459594 |
| *Clostridiaceae* | 0.00180552658031 | 0.00742029700154 | 0.00202173459518 | 0.00809073150378 |
| *Christensenellaceae* | 0.000128966184308 | 0.00484571471921 | 0.00924763403949 | 0.00254139245548 |
| norank_o_RF39 | 0.00530278604889 | 0.00479355927703 | 0.00109621256662 | 0.00545071784854 |
| *Peptococcaceae* | 0.000811728336526 | 0.0095112560927 | 0.00240104690197 | 0.00281449731637 |
| *Staphylococcaceae* | 0.000421036660534 | 0.000194397557229 | 0.00512830238777 | 0.00303070533123 |
| *Anaerovoracaceae* | 0.00127448935081 | 0.00306768828115 | 0.00137690367364 | 0.000883797674816 |
| norank_o__*Clostridia*_vadinBB60_group | 0.000713107136761 | 0.00255561666698 | 0.00112655755116 | 0.00101276385912 |
| *Tannerellaceae* | 0.00396381360593 | 0.000365088095283 | 0.00037551918372 | 0.000288277353159 |
| *Acholeplasmataceae* | 0.00465036888122 | —— | 0.000106207445901 | 0.000159311168851 |
| others | 0.00788969598119 | 0.00572287442866 | 0.0112769548808 | 0.0116486809415 |

**Table S5** The relative percentages of the gut microbiota at the genus level

| name | CON | HFD | SE-PPL | SE-PPH |
|---|---|---|---|---|
| norank_f__*Muribaculaceae* | 0.366009824189 | 0.0251901302938 | 0.00405864168263 | 0.0122100631555 |
| unclassified_f__*Lachnospiraceae* | 0.036266049652 | 0.137876230394 | 0.126090997022 | 0.0912359891517 |
| *Lactobacillus* | 0.0979801619664 | 0.0540406243481 | 0.120473381759 | 0.0949646291274 |
| *Enterococcus* | 0.00118724752025 | 0.107227796006 | 0.104011227644 | 0.122961670491 |
| *Faecalibaculum* | 0.00944108331595 | 0.0119331651715 | 0.126132721376 | 0.104250194398 |
| *Lachnospiraceae*_NK4A136_group | 0.0537902782256 | 0.0682724220987 | 0.0717962334288 | 0.047277485918 |
| *Enterorhabdus* | 0.0700248449561 | 0.0459460997212 | 0.0532706203653 | 0.033083619398 |
| *Desulfovibrio* | 0.122324425816 | 0.0190680296622 | 0.0182259563412 | 0.0276404877956 |
| *Lactococcus* | 0.000622072183132 | 0.0331708612286 | 0.0734158969788 | 0.0763631536025 |
| *Blautia* | 0.00158931856544 | 0.0244846094032 | 0.0505775029871 | 0.0608075559012 |
| norank_f__norank_o__*Clostridia*_UCG-014 | 0.016151118023 | 0.0166973277448 | 0.0526347544901 | 0.0164697403607 |
| norank_f__*Lachnospiraceae* | 0.00845866444137 | 0.0403057257193 | 0.0211542473496 | 0.0277277296262 |
| *Lachnoclostridium* | 0.00459726515827 | 0.0484836990536 | 0.0100062688531 | 0.0320405105544 |
| norank_f__*Oscillospiraceae* | 0.00543933847934 | 0.0581258178922 | 0.00714624395988 | 0.00850038879511 |
| *Colidextribacter* | 0.00287898040852 | 0.0369601911734 | 0.00677072467616 | 0.0178087128037 |
| *Alistipes* | 0.0115045422649 | 0.0173307792971 | 0.0123124774783 | 0.0116866121721 |
| *Bacteroides* | 0.0237715022664 | 0.0132493788761 | 0.00574278832477 | 0.00417243537467 |
| *Odoribacter* | 0.0109886775276 | 0.0238132266201 | 0.00261725491684 | 0.0055265803099 |
| *Romboutsia* | 0.0000531037229503 | 0.0011569025357 | 0.00614485936996 | 0.034536385533 |
| *Streptococcus* | 0.00179414721111 | 0.00996453429932 | 0.0133593794451 | 0.0155062871015 |
| *Helicobacter* | 0.0128321353386 | 0.00916039220893 | 0.00717658884443 | 0.010340053483 |
| *Candidatus_Saccharimonas* | 0.00554175280217 | 0.00463140326588 | 0.0111138505889 | 0.0152407684867 |
| norank_f__*Desulfovibrionaceae* | 0.0000417243537467 | 0.00103552259753 | 0.000728277962903 | 0.0343277637643 |

| | | | | |
|---|---|---|---|---|
| *Rikenellaceae*_RC9_gut_group | 0.0048172662962 | 0.0187835454321 | 0.00539002787945 | 0.00388036489844 |
| *Rikenella* | 0.0118307508487 | 0.0114855766495 | 0.00225690822539 | 0.00725245130578 |
| *Akkermansia* | 0.0220645968858 | 0.0000265518614751 | 0.00312174028486 | 0.00277656608569 |
| norank_f__*Ruminococcaceae* | 0.00513209551084 | 0.0108483319741 | 0.00500312932653 | 0.00513209551084 |
| *Alloprevotella* | 0.0125286854932 | 0.00554933904831 | 0.000341381076109 | 0.00208242456426 |
| *Mucispirillum* | 0.00191932027235 | 0.0142924877198 | 0.000352760445313 | 0.0030344984543 |
| *Roseburia* | 0.0013579380583 | 0.014417660781 | 0.000701727767557 | 0.00310277466952 |
| *Tuzzerella* | 0.000159311168851 | 0.0121000625865 | 0.00101655698219 | 0.00190794090314 |
| *Dubosiella* | 0.0124717886472 | 0.0000113793692036 | 0.0000151724922715 | 0.00000379312306788 |
| *Parvibacter* | 0.0118231646026 | 0.0000531037229503 | 0.00000379312306788 | 0.0000341381076109 |
| others | 0.0526068238284 | 0.104307091244 | 0.0769359151858 | 0.0661141350731 |
